# Malay Validation of Copenhagen Psychosocial Work Environment Questionnaire in Context of Second Generation Statistical Techniques

**DOI:** 10.1155/2020/7680960

**Published:** 2020-02-03

**Authors:** Ahmad Shahrul Nizam Isha, Muhammad Umair Javaid, Amir Zaib Abbasi, Sobia Bano, Muhammad Zahid, Mumtaz Ali Memon, Umair Rehman, Matthias Nübling, Asrar Ahmed Sabir, Saif Ur Rehman, Nazish Imtiaz

**Affiliations:** ^1^Department of Management & Humanities, Universiti Teknologi PETRONAS, Seri Iskandar, Malaysia; ^2^Department of Management Sciences, Lahore Garrison University, Lahore, Pakistan; ^3^Faculty of Management Sciences, SZABIST, Islamabad, Pakistan; ^4^Department of Management Sciences, GIFT Business School, GIFT University, Pakistan; ^5^Department of Management Sciences, City University of Science and IT, Peshawar, Pakistan; ^6^NUST Business School, National University of Sciences and Technology (NUST), Islamabad, Pakistan; ^7^Human Optimization and Modelling Lab, University of Waterloo, Canada; ^8^Freiburg Research Centre for Occupational Sciences, Germany; ^9^Department of Management Sciences, National Textile University, Pakistan

## Abstract

Psychosocial hazards present in workplaces are being actively investigated by researchers from multiple domains. More research and resources are required to investigate the debilitating consequences of these hazards in the developing and underdeveloped countries where this issue remains one of grave concern. This study aims at investigating the psychometric properties of Malaysian version of Copenhagen Psychosocial Questionnaire for reliability and validity purpose. The Malaysian version of COPSOQ is a multidimensional questionnaire; it comprises of 7 major formative constructs and 28 variables with an additional inclusion of two variables which are organizational loyalty and physiological health biomarkers (blood pressure and body mass index) that explicate a reflective construct which has 93 items all catering to assess psychosocial determinants present in workplace environments. Each formative second-order construct is further categorized into different reflective first-order constructs. The focus of this study was only on first-order reflective constructs. Probability sampling was used for data collection from 300 respondents working in industries with a response rate of 100%; structural equation modeling technique was applied for data analysis. All psychometric analysis performed on reflective constructs gave reliable results which demonstrate the validity of Bahasa Melayu (BM-COPSOQ) and its comprehensiveness of including relevant dimensions particularly in context to Asian region. The BM-COPSOQ will fill up the knowledge gap and provide a bridge between researchers, work professionals and practitioners, and many other workplaces for the best understanding of psychosocial work environment.

## 1. Background

Work, regardless of its nature involves certain occupational risks. In the backdrop of rapidly changing economic circumstances, risks present in workplace settings are becoming more apparent. Previously, biological, biomechanical, chemical, and radiological risks were mostly considered critical however in recent times psychosocial risks have also garnered serious attention of researchers. The developing and underdeveloped countries have displayed lack of awareness towards these emerging risks and much research needs to be done to tackle these perils in workplace settings [1].

Petrochemical industries by virtue have hazardous work environments that effect the physical and psychosocial condition of its employees. The Malaysian petrochemical industries are labor-intensive industry that presents many environmental, societal, and occupational risks. The safety, health, and wellbeing of the employees must be a priority in such high stress domains and managed prudently by different stakeholders [2].

**Figure 1:**
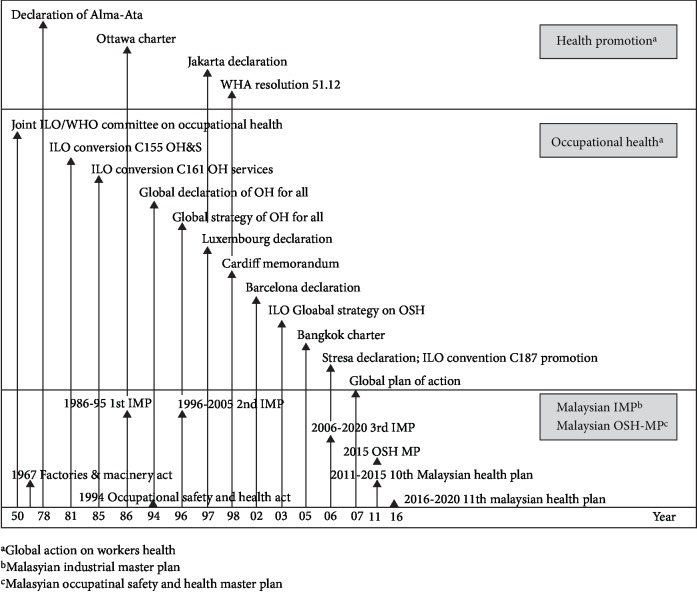
Action plan on workers health.

Malaysia has put a great emphasis on the Occupational Safety and Health (OSH) policy by enacting laws and encouraging best practice guidelines. Different organizations and awareness bodies are active in OSH domain such as Department of Occupational Safety and Health (DOSH); National Institute of Occupational Safety and Health (NIOSH); Social Security Organization (SOCSO) etc. ensuring employees maintain workplace safety standards. Moreover, International Labour Organization (ILO) and World Health Organization (WHO) have devised different action plans over the years to ensure workplaces can achieve optimal safety.  Figure 1 represents different OSH activities carried out around the world and in Malaysia.

## 2. Introduction

The psychosocial work environment possesses certain risk factors named as psychosocial risk, psychosocial hazards, psychosocial factors, or stressors (often interchangeable) with a significant deleterious effect as highlighted in many recent past studies [2–18]. These risks are an important area of inquiry precondition to creating healthy workplace environment by striving towards the maximization of workers health and wellbeing as emphasized by international agencies and organizations like International Labour Organization (ILO), World Health Organization (WHO), European Union Occupational Safety and Health (EU-OSHA) agency, Health and Safety Executive (HSE), and many others.

The Copenhagen Psychosocial Questionnaire (COPSOQ) is the best available research instrument to identify the psychosocial work environment. The value of this instrument can be supported with the number of validation studies conducted over the years by researchers internationally such as in Denmark [9], Germany [12, 13], Australia [6], Portugal [17], Spain [11], France [7], Iran [16], Chile [19], China [20], Sweden [3]; and Poland [21].

Majority of the validation studies conducted in the western countries used first generation statistical techniques with limited in-depth analysis. The studies conducted in eastern countries are also limited. In fact, there is no validation study of COPSOQ in the Malaysian context which has used for robust statistical techniques. Therefore, the aim of this research article is to present the Malaysian version of COPSOQ by analyzing the psychometric properties of the instrument. Structural Equation Modeling (SEM), a second generation statistical techniques is used in this study for robustness findings of Behasa Melayu (BM) validated version of COPSOQ.

## 3. Methodology

### 3.1. Participants

The technical workers are classified as executives and nonexecutives. These workers worked in operational, maintenance, and production activities of the petrochemical industries of Peninsular Malaysia under the leading chemical group which makes a total population of 3523. Initially, 300 total responses were collected, but due to missing values in some of the responses, the responses of 277 subjects were used in the study. From the final 277 subjects, 210 were male and 67 were female aged between 20 and 49 years. All the participants were healthy. Exclusion criteria included the use of illicit drugs, use of any prescribed medication, physical activity practice of more than five consecutive hours without having a leisure break of not less than thirty minutes of duration, provided 8 × 5 h per week, pregnant women, or women having any disturbance in regular menstrual cycles and ovulation.

### 3.2. Sampling Design

Multi-stage sampling was used due to large inquiries extending to the considerable large geographical area. The first stage in multi-stage sampling is to select the large primary sampling units like states, then areas and finally people within the selected areas [22]. Javaid et al. in their study have proposed the multi-stage sampling procedure for the petrochemical industries of Malaysia, which this study followed [1, 2].

#### 3.2.1. Selection of States

The petrochemical industries in Peninsular Malaysia are located in the states of Johor Bahru, Kedah, Pahang, and Terengganu. The study targeted petrochemical companies, which owns 80% of the shares of petrochemical industries either in the form of fully owned shares or in joint association with other petrochemical Multinational Companies (MNCs) operating in Malaysia. One fully owned and one partially owned industry were selected from each state. Therefore, Johar Bahru state was dropped because of not meeting the selection criteria.

#### 3.2.2. Selection of Petrochemical Industries from the Three Selected States

The states of Kedah, Pahang, and Terengganu were selected to represent petrochemical industries. Kedah state represents only one joint venture and only one fully owned petrochemical industry, therefore, it sets the base criteria for equal representation of the industries from the other two states, i.e., choosing one joint venture and one fully owned industry from Terengganu and Pahang respectively. Following the lottery method technique [22, 23] the names of the petrochemical industries were put in a jar, thoroughly mixed, and the required sample, which is one joint venture and one fully owned firm, from Terengganu and Pahang was randomly drawn. First, the joint venture and fully owned industries in Terengganu were added in the lottery technique followed by Pahang industries that were entered and selected based on the lottery technique.

#### 3.2.3. Selection of Study Subjects from Petrochemical Industries

The Simple Random Sampling technique was used to collect data from the provided list of the study subjects. The subjects in three industrial zones were equally divided, which means that 50 subjects from each petrochemical industry were chosen as shown in Table 1. To have a maximum representation of subjects from each targeted industry, both morning and evening shifts were targeted from the provided list. Then, 25 subjects were randomly selected from the morning shift and the remaining 25 subjects were selected from the evening shift from the selected industries. The data from all three industrial zones were collected during normal working days over a period of one month, May 2016.

### 3.3. Questionnaire

The study constructs were adapted from the second version of COPSOQ II [24], a thorough questionnaire that covers all the aspects that are important to study the psychosocial work environment along with health and wellbeing [16, 25].

#### 3.3.1. Domains of Questionnaire

The BM-COPSOQ consists of 7 different domains. Details of each domain along with relevant specifications are presented in Table 2.

#### 3.3.2. Translation of Questionnaire

The current study was conducted in Bahasa Melayu (BM) the national language of Malaysia; therefore, all the study variables were translated into BM from English using the back translation technique [26]. The forward-then-back translation procedure was completed in multiple steps. Translation and back translation of the internationally recognized base questionnaire into BM were carried out with the help of two certified translators located in Kuala Lumpur, Malaysia. In the first step, the English version was translated into BM by one certified translator, and, in the second step, the back translation from BM to English was done by another certified translator. To retain the originality and authenticity of both translations, the two selected translators (unknown to each other) worked independently. To ensure that the contents of each item were cross-linguistically comparable and generated the same meaning, the researchers used both translated languages in a single questionnaire.

#### 3.3.3. Quantitative Demands

Quantitative Demands (QD) was measured by a 4-item scale, coded by QD1, QD2, QD3, QD4, having items like “does your workload pile up due to uneven distribution?” translated into BM *“Adakah beban kerja anda semakin bertimbun disebabkan pembahagian tidak sekata?”*

#### 3.3.4. Work Pace

Work pace (WP) was measured by a 4-item scale, coded by WP1, WP2, WP3, WP4 having items like “do you have to work very fast?” translated into BM *“Adakah anda perlu bekerja dengan sangat cepat?”*

#### 3.3.5. Emotional Demands

Emotional demands (ED) was measured by a 4-item scale, coded by ED1, ED2, ED3, ED4 having items like “do you have to deal with (or manage) other people's personal problems as part of your work?” translated into BM *“Adakah anda perlu berdepan dengan (atau mengurus) masalah peribadi orang lain semasa anda bekerja?”*

#### 3.3.6. Influence at Work

Influence at work (IW) was measured by a 4-item scale, coded by IW1, IW2, IW3, IW4 having items like “Do you have a large degree of influence on the decisions concerning your work?” translated into BM *“Adakah anda mempunyai pengaruh yang kuat terhadap keputusan-keputusan yang melibatkan kerja anda?”*

#### 3.3.7. Possibilities for Development (Skill Discreation)

Possibilities for development (PD) was measured by a 4-item scale, coded by PD1, PD2, PD3, PD4 having items like “Does your work require you to take initiative?” translated into BM *“Adakah kerja anda memerlukan anda mengambil initiatif?”*

#### 3.3.8. Commitment to Workplace

Commitment to workplace (CW) was measured by a 4-item scale, coded by PD1, PD2, PD3, PD4 having items like “Do you enjoy telling others about your place of work?” translated into BM *“Adakah anda suka bercerita kepada orang lain tentang tempat kerja anda?”*

#### 3.3.9. Meaning of Work

Meaning of work (MW) was measured by a 3-item scale, coded by MW1, MW2, MW3 having items like “Is your work meaningful?” translated into BM *“Adakah kerja anda bermakna?”*

#### 3.3.10. Predictability

Predictability (PR) was measured by a 4-item scale, coded by PR1, PR2, PR3, PR4 having items like “At workplace are you informed well in advance concerning important decisions, e.g., changes or plans for future?” translated into BM *“Di tempat kerja, adakah anda dimaklumkan awal-awal lagi mengenai keputusan penting, misalnya, pertukaran atau perancangan masa hadapan?”*

#### 3.3.11. Rewards (Recognition, Prospect, Wage)

Rewards (R) was measured by a 3-item scale, coded by R1, R2, R3 having items like “Is your work recognized and appreciated by the management?” translated into BM *“Adakah kerja anda diiktiraf dan dihargai oleh pihak pengurusan?”*

#### 3.3.12. Role Clarity

Role Clarity (RC) was measured by a 3-item scale, coded by RC1, RC2, RC3 having items like “Do your work have clear objectives?” translated into BM *“Adakah kerja yang anda lakukan mempunyai objektif yang jelas?”*

#### 3.3.13. Role Conflicts

Role Conflicts (RCN) was measured by a 4-item scale, coded by RCN1, RCN2, RCN3, RCN4 having items like “Do you do things at work which are accepted by some people but not by others?” translated into BM *“Adakah anda membuat kerja yang dapat diterima oleh sesetengah orang tetapi bukan yang lain?”*

#### 3.3.14. Quality of Leadership

Quality of Leadership (QL) was measured by a 4-item scale, coded by QL1, QL2, QL3, QL4 having items like “Makes sure that each staff has good development opportunities?” translated into BM *“Memastikan setiap kakitangan baik mendapat peluang kemajuan kerjaya?”*

#### 3.3.15. Social Support Colleagues

Social Support Colleagues (SSC) was measured by a 3-item scale, coded by SSC1, SSC2, SSC3 having items like “How often your colleagues help and support you, if needed?” translated into BM *“Berapa kerapkah (rakan sekerja) anda Membantu dan menyokong anda, jika diperlukan?”*

#### 3.3.16. Social Support Supervisor

Social Support Supervisor (SSS) was measured by a 3-item scale, coded by SSS1, SSS2, SSS3 having items like “How often your immediate supervisor helps and supports you, if needed?” translated into BM *“Berapa kerapkah penyelia anda Membantu dan menyokong anda, jika diperlukan?”*

#### 3.3.17. Sense of Community/Social Community at Work

Sense of Community (SC) was measured by a 3-item scale, coded by SC1, SC2, SC3, SC4 having items like “Is there a good atmosphere between you and your colleagues?” translated into BM *“Adakah wujud suasana persekitaran yang baik antara anda dan rakan sekerja?”*

#### 3.3.18. Job Insecurity

Job Insecurity (JI) was measured by a 4-item scale, coded by JI1, JI2, JI3, JI4 having items like “are you worried about becoming unemployed?” translated in BM *“Adakah anda risau tentang menjadi penganggur?”*.

#### 3.3.19. Work-Family Conflict

Work-Family Conflict (WFC) was measured by a 4-item scale, coded by WFC1, WFC2, WFC3, WFC4 having items like “Do you feel that your work drains so much of your energy that it has a negative effect on your personal life?” translated into BM “*Adakah anda berasa yang kerja menghabiskan begitu banyak tenaga anda sehingga ia mempunyai kesan negatif ke atas kehidupan peribadi?”.*

#### 3.3.20. Family-Work Conflict

Family-Work Conflict (FWC) was measured by a 2-item scale, coded by FWC1, FWC2 having items like “Do you feel that your personal life takes so much of your energy that it has a negative effect on your work?” translated into BM *“Adakah anda berasa yang kehidupan peribadi anda mengambil begitu banyak tenaga sehingga mempunyai kesan negatif ke atas pekerjaan anda?”*

#### 3.3.21. Trust

Trust (T) was measured by a 7-item scale, coded by T1, T2, T3, T4, T5, T6, T7 having items like “Do the employees withhold information from each other?” translated in BM *“Adakah pekerja merahsiakan maklumat antara satu sama lain?”*

#### 3.3.22. Justice and Respect

Justice and Respect (JR) was measured by a 4-item scale, coded by JR1, JR2, JR3, JR4 having items like “Are conflicts resolved in a fair way?” translated in BM *“Adakah konflik diselesaikan dengan cara adil?”*

#### 3.3.23. General Health

General Health (GH) was measured by a 1-item scale, coded by GH1 having item “In general, how would you rate your health?” translated in BM *“pada amnya, bagaimanakah anda kadar kesihattan anda?”*

#### 3.3.24. Sleeping Trouble

Sleeping Trouble (ST) was measured by a 4-item scale, coded by ST1, ST2, ST3, ST4 having items like “How often have you slept badly and restlessly?” translated in BM *“Berapa kerapkah anda tidak dapat tidur dengan lena dan nyenyak?”*

#### 3.3.25. Burnout

Burnout (BO) was measured by a 4-item scale, coded by BO1, BO2, BO3, BO4 having items like “How often have you felt worn out?” translated in BM *“Berapa kerapkah anda berasa lesu?”*

#### 3.3.26. Stress

Stress (STR) was measured by a 4-item scale, coded by STR1, STR2, STR3, STR4 having items like “How often have you had problems relaxing?” translated in BM *“Berapa kerapkah anda mempunyai masalah untuk berehat?”*

#### 3.3.27. Job Satisfaction

Job Satisfaction (JS) was measured by a 4-item scale, coded by JS1, JS2, JS3, JS4 having items like “Regarding your work in general how pleased are you with your work prospects?” translated in BM *“Berkenaan kerja anda pada keseluruhannya, adakah anda puas hati dengan prospek pekerjaan anda?”*

#### 3.3.28. Organizational Loyalty

Organizational Loyalty (OL) was measured by a 3-item scale, coded by OL1, OL2, OL3 having items like “I sometimes feel like leaving this employment for good?” translated in BM *“Saya kadang-kadang terasa seperti hendak meninggalkan pekerjaan ini untuk selamanya?”*

#### 3.3.29. Biomarker – Blood Pressure

Blood Pressure (BP) was measured as per practice guidelines of the European society of hypertension [27, 28]. The mean arterial blood pressure **(MAP)** is defined as average blood pressure in an individual during a single cardiac cycle as shown in the following equation:

(1) $$\begin{array}{c} MAP=SBP+\frac{2\left(DBP\right)}{3}. \end{array}$$

In this equation, SBP is the systolic blood pressure and DBP is the diastolic blood pressure. The unit of mean arterial pressure (MAP) is measured in mmHg. MAP is used to approximate the pressure gradient (ΔP) of the subjects and includes the effect of systolic and diastolic pressure. Measurements from left arm was taken and measured as “Systolic blood pressure reading 1, left arm” and “Diastolic blood pressure reading 1, left arm,” translated in BM as “*Tekanan darah sistolik Bacaan 1, Tangan kiri”* and “*Tekanan darah diastolik Bacaan 2, Tangan kiri*,” respectively. Similarly, for right arm it was measured as “Systolic blood pressure reading 2, right arm” and “Diastolic blood pressure reading 2, right arm,” translated in BM as “*Tekanan darah sistolik Bacaan 2, Tangan kanan”* and “*Tekanan darah diastolik Bacaan 2, Tangan kanan*,” respectively.

#### 3.3.30. Biomarker – Body Mass Index

WHO BMI index is defined as weight in kilograms divided by the square of the height in meters (kg/m^2^). BMI was calculated with the following formula:

(2) $$\begin{array}{c} BMI\left[kg/m^{2}\right]=\frac{\mathrm{Weight}\mathrm{in}\mathrm{Kilometers}}{\text{Height in Meters}\times \text{Height in Meters}}. \end{array}$$

BMI as endogenous non-invasive biomarker variable was calculated with a single item. Weight was measured in kilograms “How much do you weigh?” translated into BM *“Berapakah berat anda?”* Height was measured in meters “How tall are you?” translated into BM *“Berapakah ketinggian anda?”*

### 3.4. Statistical Approach

In this study the complexity of the model is high and therefore we have used Partial Least Squares Structural Equation Modeling (PLS-SEM) technique to evaluate the psychometric properties of Copenhagen psychosocial work environment questionnaire [29]. They further argued, a model involving reflective and formative constructs is reflected as a multifaceted measurement model. Copenhagen scale consists of reflective constructs on first-order level and formative constructs on second-order level. For instance, demands at work is a second-order formative construct which is based on three first-order reflective constructs such as emotional demands, work pace, and quantitative demands as shown in Table 2. However, in this study, we only aimed to examine the first-order reflective constructs for psychometric properties via applying the second generation tool, i.e., PLS-SEM. The first-order reflective constructs of Copenhagen are discussed in Table 2 under dimensions section.

In order to examine the quality of reflective constructs in terms of reliability and validity, we used the following standards as suggested that the item loading should be greater than 0.60 or at minimum value of 0.40, the Cronbach's alpha and composite reliability are to be at 0.70 or greater, and the convergent validity also termed average variance extracted (AVE) must exceed the value 0.50.

Another criterion to examine the reflective construct is discriminant validity which is defined as to what extent each latent variable is different from other variables in a study model. Few authors added that the AVE of each variable must be greater than the highest squared correlation of variables with any other latent variables in the model to form discriminant validity [29, 30].

The study analyses were conducted using WarpPLS 6.0 [31] software as it provides us many options for the assessment of outer model parameters and calculating the latent variables scores (LVs). Using WarpPLS 6.0, we performed the algorithm, i.e., PLS regression for outer model to assess the first-order reflective measurement (outer) model parameters and LVs. The results of first-order reflective measurement model comprising factor loadings, Cronbach's alpha, composite reliability (CR), AVE, and discriminant validity are discussed in Tables 3 and 4, respectively.

### 3.5. Results

Using the WarpPLS, we assessed the first-order reflective dimensions for their reliability and validity in terms of indicator loadings, Cronbach's alpha, composite reliability, convergent validity, and discriminant validity. The results shown in Table 3 reported that the indicator loadings have exceeded the critical value of 0.40 to retain an item. However, few items did not meet threshold value of 0.40, for instance, QD4 of quantitative demands and T1, T2, and T6 of trust variable. Next, we checked the reliability tests comprising Cronbach's alpha and composite reliability and found that all constructs are reliable as they met the critical value of 0.70. We also evaluated the convergent validity via using the criteria of AVE and resulted that all reflective constructs have achieved the AVE value of 0.50, hence verifying that all constructs had met the requirement of convergent validity, see Table 3.

Another criteria to examine the reflective constructs of BM-COPSOQ, is by means of discriminant validity. While assessing the discriminant validity, we compared the square root of AVE with the correlation of latent variables. As a result, Table 4 showed that there is no discriminant validity issue as the square root of AVE of all constructs is greater than the correlation of other variables as shown in Table 4.

We also calculated the full Collinearity (FVIF) which refers to the vertical and lateral Collinearity of one construct in association with other variables [32]. They further suggested that FVIF is another source to establish the discriminant validity and the critical value of FVIF should be equal to or less than five. As shown in Table 3, we have found that none of FVIF is greater than the threshold value.

## 4. Discussion and Conclusion

We have evaluated the psychometric properties of BM-COPSOQ using a sample of Malaysian Petrochemical Industry workers with the help of probability sampling for data gathering and SEM technique for data analysis. Main goal of this study was to provide a valid and reliable psychosocial work environment questionnaire for an eastern industrially developing country Malaysia in Bahasa Melayu.

The BM-COPSOQ is a standardized self-report measure which is comprised of 7 constructs having 28 variables with 93 items designed for the assessment of psychosocial workplace environment. The details of each construct followed by variables and number of items are already summarized in aforementioned Table 2. The 4 items, i.e., one from the “quantitative demands” and 3 from “trust” were dropped due to low factor loadings. The composite reliability of all the items is well above 0.7 so as average variance extracted which is more than 0.5 for all variables.

We have added a construct “further parameter” having two variables in it which are “organizational loyalty” and “biomarkers” Organizational loyalty is measured by three items while biomarker is made up of blood pressure and body mass index which are physiological health measures. The details of measurement of each physiological variable are already explained in Sections 3.3.29 and 3.3.30. Organizational loyalty (Intention to leave) is predicted as another outcome of working condition which results from psychosocial hazards.

Several strengths of this study need to be highlighted. Firstly, the inclusion of two physiological health biomarkers (BP and BMI). In behavioral studies, the use of biomarkers in context to the psychosocial work environment factors is largely lacking. The inclusion of two non-invasive health biomarkers was used as a screening tool to measure the physiological health of the workers besides psychological such as stress and burnout in the psychosocial work environment. The screening tools will help workers to keep updated with their health conditions due to psychosocial risks emerged around them. Secondly, the use of SEM for evaluating the psychometric properties of BM-COPSOQ and to ensure the robustness of the results which eventually we have found by achieving the validity and reliability of the items well above their minimum cut off values. Thirdly, the probability sampling technique to reach out the technical samples working in different states of highly hazardous petrochemical industries ensured greater confidence in results.

There were some limitations to this study. Firstly, the study sample is based on workers working only in the petrochemical industries of Peninsular Malaysia which should be expanded to different regions, branches and professions in the country. Secondly, this study is limited to only evaluate the psychometric properties of BM-COPSOQ. Our upcoming research article focuses on COPSOQ III where we have thoroughly evaluated the impact of all the higher-order constructs such as Demands at Work; Work Organization and Job Content; Interpersonal Relations and Leadership; Work-Individual Interference; & Health and Wellbeing (inclusive of health non-invasive biomarkers) in lieu to CORE and additional items. These higher-order constructs will be used as reflective-formative constructs to widen the scope of COPSOQ [33, 34].

## Figures and Tables

**Table 1:** No. of respondents per industry.

*States*	*Petrochemical industry category*		*Total respondents*
	Fully owned	Joint venture	
*Kertih, Terengganu*	50	50	100
*Gebeng, Pahang*	50	50	100
*Kedah, Gurun*	50	50	100

*Total respondents*	150	150	300

**Table 2:** Total Domains and Dimensions of BM‐COPSOQ.

*Domains with total items*	*Dimensions*	
Demands at work (12 items) *Desakan di tempat kerja*	Quantitative demands *“QD” 4 items*	Keperluan Kuantitatif
	Work pace “*WP*” *4 items*	Kadar Bekerja
	Emotional demands “*ED*” *4 items*	Keperluan Emosi
Work organization and job contents (15 items) *Penyusunan kerja Dan Kandungan Kerja*	Influence at work “IW” *4 items*	Pengaruh di tempat kerja
	Possibilities for development “*PD*” *4 items*	Potensi untuk Pembangunan
	Commitment to workplace “*CW*” *4 items*	Komitmen terhadap tempat kerja
	Meaning of work “MW” *3 items*	Kerja Bermakna
Interpersonal relations and leadership (27 items) *Hubungan Antara Perorangan Dan Kepimpinan*	Predictability “*PR*” *4 items*	Seperti yang dijangkakan
	Recognition (rewards) “*R*” *3 items*	Pengiktirafan (Ganjaran)
	Role clarity “*RC*” *3 items*	Kejelasan Peranan
	Role conflicts “*RCN*” *4 items*	Konflik Peranan
	Quality of leadership “*QL*” *4 items*	Kualiti Kepimpinan
	Social support supervisor “*SSS*” *3 items*	Sokongan Sosial penyelia
	Social support colleagues “*SSC*” *3 items*	Sokongan Sosial rakan sekerja
	Sense of community “*SC*” (social community at work) *3 items*	Perasaan Kemasyarakatan (komuniti sosial di tempat kerja
Work‐individual interface (14 items) *Hubung Kait Individu‐Kerja*	Job insecurity “*JI*” *4 items*	Keadaan pekerjaan yang tidak terjamin
	Job satisfaction “*JS*” *4 items*	Kepuasan Bekerja
	Work‐family conflict “*WFC*” *4 items*	Konflik Pekerjaan dan Keluarga
	Family‐work conflict “*FWC*” *2 items*	Konflik keluarga dan pekerjaan
Values at workplace level (11 items) *Nilai di tempat kerja*	Trust “*T*” *7 items*	Kepercayaan
	Justice and respect “*JR*” *4 items*	Keadilan dan Penghormatan
Health and wellbeing (13 items) *Kesihatan dan Kesejahteraan*	General health “*GH*” *1 item*	Kesihatan am
	Sleeping trouble “*ST*” *4 items*	Masalah tidur
	Burnout “*BO*” *4 items*	Kehabisan tenaga
	Stress “*STR*” *4 items*	Tekanan

*Further parameters*		
Organizational Loyalty *Niat untuk berhenti*	Organizational loyalty “*OL*” (intention to leave) *3 items*	Kesetiaan kepada Organisasi (Niat untuk berhenti)
Biomarker *Biomarker*	Blood Pressure “*BP*” *1 item*	Tekanan Darah
	Body Mass Index “*BMI*” *1 item*	Indeks Jisim Badan

**Table 3:** Constructs reliability and validity.

Constructs	Construct‐items	Loadings	CR	CronBach	AVE	FVIF
*Demands at work*	QD1	0.865	0.909	0.85	0.77	1.597
	QD2	0.898				
	QD3	0.868				
*Work pace*	WP1	0.827	0.927	0.895	0.761	1.541
	WP2	0.883				
	WP3	0.909				
	WP4	0.869				
*Emotional demands*	ED1	0.747	0.869	0.799	0.625	2.295
	ED2	0.748				
	ED3	0.850				
	ED4	0.813				
*Influence at work*	IW1	0.650	0.817	0.701	0.53	1.397
	IW2	0.710				
	IW3	0.732				
	IW4	0.811				
*Possibilities for development*	PD1	0.663	0.830	0.727	0.551	1.421
	PD2	0.767				
	PD3	0.789				
	PD4	0.744				
*Commitment to workplace*	CW1	0.844	0.833	0.731	0.559	1.297
	CW2	0.756				
	CW3	0.762				
	CW4	0.608				
*Meaning of work*	MW1	0.835	0.879	0.792	0.707	1.56
	MW2	0.893				
	MW3	0.793				
*Predictability*	PR1	0.789	0.823	0.711	0.544	1.321
	PR2	0.833				
	PR3	0.758				
	PR4	0.534				
*Recognition*	R1	0.886	0.924	0.876	0.802	2.478
	R2	0.917				
	R3	0.882				
*Role clarity*	RC1	0.825	0.901	0.834	0.752	1.599
	RC2	0.929				
	RC3	0.844				
*Role conflicts*	RCN1	0.669	0.852	0.767	0.592	1.334
	RCN2	0.803				
	RCN3	0.855				
	RCN4	0.738				
*Quality of leadership*	QL1	0.812	0.913	0.873	0.725	2.225
	QL2	0.871				
	QL3	0.850				
	QL4	0.871				
*Social support colleagues*	SSC1	0.899	0.919	0.868	0.791	1.906
	SSC2	0.900				
	SSC3	0.869				
*Social support supervisor*	SSS1	0.943	0.957	0.932	0.88	2.217
	SSS2	0.944				
	SSS3	0.927				
*Sense of community*	SC1	0.890	0.914	0.858	0.779	1.951
	SC2	0.910				
	SC3	0.847				
*Job insecurity*	JI1	0.810	0.865	0.791	0.615	1.175
	JI2	0.746				
	JI3	0.814				
	JI4	0.766				
*Job satisfaction*	JS1	0.741	0.907	0.862	0.711	1.882
	JS2	0.823				
	JS3	0.878				
	JS4	0.920				
*Work‐family conflict*	WFC1	0.712	0.905	0.857	0.707	2.866
	WFC2	0.905				
	WFC3	0.930				
	WFC4	0.797				
*Family‐work conflict*	FWC1	0.960	0.959	0.915	0.922	1.992
	FWC2	0.960				
*Trust*	T3	0.640	0.838	0.741	0.566	2.314
	T4	0.827				
	T5	0.758				
	T7	0.773				
*Justice and respect*	JR1	0.771	0.865	0.791	0.617	2.982
	JR2	0.796				
	JR3	0.844				
	JR4	0.726				
*General Health*	GH1	1.000	1	1	1	1.171
*Sleeping trouble*	ST1	0.755	0.918	0.879	0.738	1.672
	ST2	0.874				
	ST3	0.877				
	ST4	0.921				
*Burnout*	BO1	0.917	0.947	0.925	0.817	2.379
	BO2	0.903				
	BO3	0.873				
	BO4	0.922				
*Stress*	STR1	0.582	0.892	0.833	0.679	2.798
	STR2	0.870				
	STR3	0.901				
	STR4	0.899				
*Organizational loyalty*	OL1	0.879	0.854	0.74	0.664	1.258
	OL2	0.869				
	OL3	0.681				
*BMI*	BMI	1.000	1	1	1	1.171
*MAP*	MAP	1.000	1	1	1	1.417

**(a):** 

	QD	WP	ED	IW	PD	CW	MW	OL	PR	R	RC	RCN	QL	SSC
QD	*0.877*													
WP	0.354	*0.872*												
ED	0.463	0.386	*0.791*											
IW	0.136	0.228	0.256	*0.728*										
PD	0.262	0.238	0.134	0.331	*0.742*									
CW	0.059	0.059	0.120	0.073	0.105	*0.747*								
MW	−0.082	0.040	−0.162	0.113	0.222	0.097	*0.841*							
OL	0.120	0.132	0.233	0.000	0.045	−0.026	−0.210	*0.815*						
PR	−0.069	0.118	0.011	0.072	0.131	0.075	0.169	−0.107	*0.737*					
R	−0.180	0.052	−0.336	0.136	0.033	0.079	0.293	−0.240	0.324	*0.895*				
RC	−0.210	−0.012	−0.212	0.058	−0.030	0.045	0.434	−0.234	0.237	0.320	*0.867*			
RCN	0.222	0.119	0.252	0.009	0.064	0.288	−0.084	0.140	−0.124	−0.222	−0.115	*0.770*		
QL	−0.142	0.133	−0.226	0.117	0.113	−0.045	0.296	−0.253	0.302	0.517	0.294	−0.140	*0.851*	
SSC	−0.229	−0.063	−0.232	0.139	0.133	0.136	0.304	−0.203	0.274	0.265	0.289	−0.046	0.337	*0.889*

**Table:** (b)

	SSS	SC	JI	JS	WFC	FWC	T	JR	GH	ST	BO	STR
SSS	*0.938*											
SC	0.375	*0.883*										
JI	−0.012	−0.178	*0.784*									
JS	0.394	0.484	−0.117	*0.843*								
WFC	−0.301	−0.233	0.263	−0.286	*0.841*							
FWC	−0.163	−0.215	0.158	−0.235	0.667	*0.960*						
T	0.473	0.424	−0.119	0.445	−0.222	−0.168	*0.752*					
JR	0.497	0.297	−0.129	0.402	−0.359	−0.251	0.665	*0.785*				
GH	0.115	0.115	−0.035	0.150	−0.168	−0.104	0.009	0.091	*1.000*			
ST	−0.219	−0.074	0.065	−0.156	0.387	0.331	−0.185	−0.218	−0.128	*0.859*		
BO	−0.180	−0.198	0.150	−0.187	0.513	0.378	−0.150	−0.248	−0.160	0.522	*0.904*	
STR	−0.255	−0.254	0.151	−0.285	0.577	0.375	−0.282	−0.335	−0.133	0.540	0.707	*0.824*

Note: The square roots of average variances extracted (AVEs) shown on diagonal with italic numbers.

## Data Availability

Data of 277 study samples used will be provided on request.
